# MicroRNAs Enable mRNA Therapeutics to Selectively Program Cancer Cells to Self-Destruct

**DOI:** 10.1089/nat.2018.0734

**Published:** 2018-09-24

**Authors:** Ruchi Jain, Josh P. Frederick, Eric Y. Huang, Kristine E. Burke, David M. Mauger, Elizaveta A. Andrianova, Sam J. Farlow, Summar Siddiqui, Jeffrey Pimentel, Kahlin Cheung-Ong, Kristine M. McKinney, Caroline Köhrer, Melissa J. Moore, Tirtha Chakraborty

**Affiliations:** ^1^ Department of Molecular Biology, Moderna Therapeutics, Cambridge, Massachusetts.; ^2^ Department of Oncology, Moderna Therapeutics, Cambridge, Massachusetts.; ^3^ New Venture Labs, Moderna Therapeutics, Cambridge, Massachusetts.; ^4^ Non-Clinical Sciences, Moderna Therapeutics, Cambridge, Massachusetts.; ^5^ Computational Sciences, Moderna Therapeutics, Cambridge, Massachusetts.; ^6^ Rare Diseases, Moderna Therapeutics, Cambridge, Massachusetts.

**Keywords:** miRNA, mRNA, therapeutic, suicide therapy, RNA modifications

## Abstract

The advent of therapeutic mRNAs significantly increases the possibilities of protein-based biologics beyond those that can be synthesized by recombinant technologies (eg, monoclonal antibodies, extracellular enzymes, and cytokines). In addition to their application in the areas of vaccine development, immune-oncology, and protein replacement therapies, one exciting possibility is to use therapeutic mRNAs to program undesired, diseased cells to synthesize a toxic intracellular protein, causing cells to self-destruct. For this approach to work, however, methods are needed to limit toxic protein expression to the intended cell type. Here, we show that inclusion of microRNA target sites in therapeutic mRNAs encoding apoptotic proteins, Caspase or PUMA, can prevent their expression in healthy hepatocytes while triggering apoptosis in hepatocellular carcinoma cells.

## Introduction

In recent years, synthetic messenger RNA has emerged as a powerful alternative to conventional DNA-based gene therapy for driving protein expression *in vivo*. Because of its transient nature and its extremely low likelihood of genome integration, mRNA mitigates two of the major risks associated with gene therapy [1]. Further, exploiting the cell's natural machinery to synthesize a therapeutic protein is likely to yield native folding and proper post-translational modifications, providing a key advantage over administration of recombinantly produced synthetic proteins.

To date, significant progress has been made in advancing the use of mRNA therapeutics for vaccine development, immune-oncology, and protein replacement. A potential therapeutic area yet to be explored, however, is the delivery of mRNAs encoding toxic intracellular proteins that selectively elicit cell death in undesired, diseased cells. Success of such a Trojan horse approach would require mechanisms to tightly limit toxic protein expression to the target cell type, which remains an obstacle for mRNA therapeutics in general [1–4]. While minimizing off-target expression would be beneficial in the development of protein replacement therapies and cancer immunotherapies, it will be vital for any approach aimed at selective elimination of cells by mRNAs encoding toxic payloads.

Most current delivery methods exhibit a high propensity for expression in the liver upon systemic administration [5], suggesting that mRNA strategies to program death of diseased cells remain unviable unless ways can be found to eliminate mRNA delivery to and/or block mRNA expression in the liver.

Here, we demonstrate that cellular microRNAs (miRNAs) with cell type- and disease-specific expression profiles can be recruited to degrade a synthetic, modified mRNA by a small interfering RNA (siRNA)-like cleavage mechanism. This strategy can be utilized to suppress protein expression from the mRNA in unintended recipient cells. The conserved principles of miRNA–mRNA regulation allow this technology to be applied across species. Exploiting endogenous miRNA pools, we are able to drive apoptosis of tumor cells using an mRNA encoding a toxic protein, while avoiding damage to the liver. Such miRNA-mediated elimination of liver toxicity is effective for both intratumoral and systemic administration, even at high doses of potentially lethal mRNAs.

## Materials and Methods

### Synthesis of modified mRNA

Plasmids with a T7 promoter site and codon-optimized reporter genes were synthesized by Atum (Menlo Park, CA). Open reading frame and untranslated regions (UTR) sequences are summarized in Supplementary Table S1 (Supplementary Data are available online at www.liebertpub.com/nat). Pforward and Preverse were used as primers to amplify all reporters (Supplementary Table S2). Polymerase chain reaction (PCR) amplifications were performed with Phusion polymerase (NEB, Ipswich, MA) following the manufacturer's guidelines, and the reaction was cleaned up using PURELINK™ PCR Micro Kit (No. K310250; Invitrogen, Waltham, MA). Resulting templates were used for *in vitro* transcription reactions using T7 RNA Polymerase (NEB).

Nucleotide triphosphate mixes were made using unmodified adenosine, cytidine, and guanosine with 1-methyl-pseudouridine (m^1^Ψ). The transcription reaction was subjected to a DNase treatment, and then purified using MEGACLEAR™ Kit (No. AM1908; Ambion, Austin, TX). Vaccinia virus capping enzyme (NEB) and 2′*O*-methyltransferase enzymes (NEB) were used per the manufacturer's instructions to introduce a 5′ cap. The RNA was purified using Ambion's MEGACLEAR Kit and analyzed on a bioanalyzer chip (Agilent 2100). For experiments in rodents and monkeys, RNA was further purified using reverse-phase chromatography.

### Cell culture

HeLa (ATCC CCL-2), RAW 264.7 (ATCC TIB-71), and Jurkat (ATCC T1B-152) cell lines were obtained from ATCC and cultured under standard conditions. Primary human hepatocytes were obtained from BioreclamationIVT (No. F0995; Westbury, NY).

### Transfection of HeLa, RAW 264.7, and primary human hepatocytes

HeLa, RAW 264.7, and primary human hepatocytes were seeded in 100-μL media per well of 96 well plates at concentrations of 1.7 × 10^4^, 4 × 10^5^, and 2 × 10^5^ cells/mL 1 day before transfection. Said amounts of mRNA were transfected with 0.3 μL of Lipofectamine 2000 (L2000) (No. 11668027; Life Technologies, Camarillo, CA) following the manufacturer's guidelines. The transfection complexes were removed after 4 h, and the cells were replenished with fresh media.

### Transfection of Jurkat cells

Jurkat cells were electroporated using the Neon Transfection System (Life Technologies; No. MPK5000) following the manufacturer's guidelines. Six hundred microliters of Jurkat cells at a concentration of 1 × 10^7^ cells/mL were electroporated with 8 μg of mRNA and added to a prepared six-well plate with 3 mL of prewarmed media.

### Luciferase assay in cells

Cells transfected with 20 ng of luciferase (Luc) mRNA were analyzed 6 h after transfection for Luc luminescence per the manufacturer's instructions (No. E4030; Promega, Madison, WI).

### Erythropoietin assay in cells

Supernatant from cells transfected with 20 ng of erythropoietin (Epo) mRNA was analyzed 6 h after transfection for Epo levels by enzyme-linked immunosorbent assay (ELISA) per the manufacturer's instructions (No. 01630; Stem Cell Technologies, Cambridge, MA).

### Quantitative reverse transcription polymerase chain reaction

For Jurkat cells, total RNA was extracted from cells using the miRNeasy Micro Kit (No. 217084, Qiagen, Valencia, CA). One hundred nanograms of RNA was reverse transcribed using the high-capacity cDNA reverse transcription kit (No. 4368817; Life Technologies). The resulting cDNA was used for quantitative reverse transcription polymerase chain reaction using SYBR Green (No. 172-5274; Biorad, Portland, ME). Luc and Epo transcripts were amplified across different amplicons with three different primer pairs for Luc (Pf1 and Pr1, Pf2 and Pr2, and Pf3 and Pr3) and one primer pair for Epo (Pf and Pr). Reporter transcript levels were normalized to HPRT transcript levels measured using human and mouse HPRT primers, HPRT_f and HPRT_ r. All primer sequences used are described in Supplementary Table S2.

For hepatocytes, total RNA was extracted from cells using the Maxwell machine from Promega and the RSC simplyRNA Tissue Kit (No. AS1340). RNA was reverse transcribed using the Taqman^™^ RNA-to-CT^™^ 1 Step-kit (No. 4392653). Taqman-validated primer–probe pairs were used, miR-122-5p (catalog No. 4427975, Assay Id No. 002245), Z30 (catalog No. 4427975, Assay Id No. 001092), ALDOA (catalog No. 4331182, Assay Id No. Hs00605108_g1), GYS1 (catalog No. 4331182, Assay Id No. Hs00157863_m1), P4HA1 (catalog No. 4331182, Assay Id No. Hs00914594_m1), CCNG1 (catalog No. 4331182, Assay Id No. Hs00171112_m1), and GAPDH (catalog No. 4331182, Assay Id No. Hs02786624_g1). miR-122-5p levels were normalized to Z30 RNA. P4HA1, ALDOA, GYS1, and CCNG1 mRNAs were normalized to GAPDH mRNA levels.

### 5′-Phosphate sequencing for detecting specific cleavage products

Ten micrograms each of total RNA samples was used for the analysis. 5′-phosphate sequencing was done as follows: Total RNA samples were ligated to the Illumina_5SR RNA adapter (NEB; No. E7328A) (5′-rGrUrUrCrArGrArGrUrUrCrUrArCrAr GrUrCrCrGrArCr GrArUrC-3′) using T4 RNA Ligase 1 (NEB; No. M0204L) following the manufacturer's guidelines. Reverse transcription was performed with an OligodT primer using Superscript III Reverse Transcriptase (Life Technologies; No. 18080-093) and RNaseOUT Recombinant Ribonuclease Inhibitor (Life Technologies; No. 10777-019).

The purified cDNA was amplified using Q5 DNA polymerase (NEB; No. M0491S) with the SR Illumina (NEB; No. E7310A, 5′ AATGATACGGCGACCACCGAGATCTACACGTTCAGAGTTCTACAG TCCG-s-A-3′) and the 3′ Oligo primer (Supplementary Table S2) targeted to the RNA's 3′ UTR. Purified PCR products were subjected to a second PCR amplification step with the SR Illumina and Illumina barcoded primers (NEB; No. E7500S). Libraries were multiplexed and sequenced using the MiSeq Instrument (Illumina). Sequencing reads were aligned to the reference RNA using the Bowtie2 program, and the locations of the 5′ ends for all aligned sequencing reads were counted using a custom Perl script.

### Lipid nanoparticle formulation

The ionizable lipid, DLin-MC3-DMA ((6Z, 9Z, 28Z, 31Z)-heptatriaconta-6, 9, 28, 31-tetraen-19-yl 4- (dimethylamino) butanoate), and DLin-KC2-DMA were synthesized as previously described [6]. 1,2-Distearoyl-sn-glycero-3-phosphatidylcholine (DSPC) and cholesterol were purchased from Avanti Polar Lipids. 1,2-Dimyristoyl-sn-glycerol, methoxypolyethylene glycol 2K (PEG-DMG) was purchased from NOF America Corporation. All other laboratory supplies and chemicals were purchased from Fisher Scientific. Lipid nanoparticle (LNP) formulations were prepared using a published method with minor modifications [7].

In brief, lipid components were dissolved in ethanol at molar ratios of 50:10:38.5:1.5 (ionizable lipid:DSPC:cholesterol:PEG-lipid). The lipid mixture was combined with a 50-mM citrate buffer (pH 4.0) containing mRNA at a volume ratio of 3:1 (mRNA:lipid) and total flow rate of 14 mL/min using a NanoAssembler system (Precision NanoSystems, Vancouver, BC). Formulations were dialyzed in 10-kDa membrane dialysis cassettes against phosphate-buffered saline (PBS, pH 7.4) for at least 18 h, followed by concentration using 100-kDa Amicon ultracentrifugal filters, filtration through a 0.22-μm filter, and storage in presterilized vials at 4°C until use. All formulations were tested for particle size, RNA encapsulation, and endotoxin. Formulations were within the following parameters: 80–100 nm average size (polydispersity <0.2), >90% encapsulation, and <1 EU/mL endotoxin.

### Rodent experiments

Animal studies were performed in accordance with the National Institutes of Health (NIH) Guide for Care and Use of Laboratory Animals, and approved by the Institutional Animal Care and Use Committee of Moderna Therapeutics. Female BALB/c mice, 8 weeks old, weighing 18–23 g, and female Sprague Dawley rats, 8 weeks old, weighing 275–300 g (Charles River Laboratories, Wilmington, MA), were prewarmed using a heating lamp before injecting in the lateral tail vein using a 1-mL syringe with a 27G 1/2′′ needle (Becton Dickson, San Diego, MA) with LNP-encapsulated 0.05 mg/kg mRNA encoding Luc or Epo or 2 mg/kg mRNA encoding Caspase. For mice studies with Epo transfected with L2000, 0.1 mg/kg mRNA was complexed with Lipofectamine 2000 in the presence of Optimem. Lipofectamine and Opti-MEM (Thermo Fisher Scientific; No. 51985091) were used at a ratio of 0.04:1.

### Nonhuman primates experiment

Study was conducted at RxGen/St. Kitts Biomedical Research Foundation according to the facility standard operating procedures. Two- to five-year-old male African green monkeys (*Chlorocebus sabaeus*), weighing 3.0–4.5 kg, were dosed with MC3-encapsulated mRNA at a dose of 0.05 mg/kg in a slow intravenous (IV) bolus over 1–2 min. Six hours' postinfusion, blood was collected from a peripheral vein and analyzed for Epo protein levels by ELISA (Stem Cell Technologies; No. 01630).

### Hep3b tumor xenograft mouse model study

Study was conducted at Molecular Imaging, and compliant with all the laws, regulations, and guidelines of the NIH and with the approval of Molecular Imaging, Inc.'s Animal Care and Use Committee. Hep 3B2.1-7 cells were expanded and implanted in 11–12 weeks old female Taconic C.B-17 SCID mice (C.B-Igh-1b/IcrTac-*Prkdcscid*). Before implant, all mice were slightly anesthetized through inhalation of isoflurane/oxygen, and the hair over the implantation site was shaved using electric clippers. Test animals were implanted subcutaneously in the right flank on day 0 with 5 × 10^6^ cells in 0.2 mL of 50% serum-free medium: 50% Matrigel^®^ using a 27-gauge needle and syringe.

All mice were sorted into study groups based on caliper measurement estimation of tumor burden on day 22 when the mean tumor burden for all animals was 394 mm^3^ (range of group means, 372–424 mm^3^). The mice were distributed to ensure that the mean tumor burden for all groups was within 10% of the overall mean tumor burden for the study population. Tris-8% Sucrose vehicle control and MC3-encapsulated 6.25 or 25 μg mRNA were dosed at a fixed volume of 25 μL.

### MC38 tumor mouse model study

Study was performed in accordance with the NIH Guide for Care and Use of Laboratory Animals, and approved by the Institutional Animal Care and Use Committee of Moderna Therapeutics. MC38 cells were expanded and implanted in 6–8-week female C57BL/6 mice from The Jackson Laboratory (C57BL/6J, No. 000664). Before implant, hair over the implantation site was shaved using electric clippers. Test animals were implanted subcutaneously in the right flank on day 0 with 5 × 10^5^ cells in 0.1 mL of PBS using a 27-gauge needle and syringe.

All mice were sorted into study groups based on caliper measurement of tumor burden on day 10 when the mean tumor burden for all animals was 175 mm^3^ (range of group means, 170–180 mm^3^). The mice were distributed to ensure that the mean tumor burden for all groups was within 10% of the overall mean tumor burden for the study population. PBS control and MC3-encapsulated mRNA were dosed at a fixed volume of 25 μL.

### Luc assay in animals

Luciferin, the substrate of Luc, was injected intraperitoneally into mice or rats at a dose of ∼150 mg/kg body weight. Twenty-minutes after luciferin injection, animals were euthanized. Whole body imaging or *ex vivo* imaging was carried out as specified. Regions of interest were drawn manually over tissues, and bioluminescent signal intensity was analyzed on the IVIS spectrum by using Living Image Software (Perkin Elmer, Waltham, MA) and expressed as photons/s/cm^2^/sr.

### Epo assay in animals

Blood was spun down at 7,000 rpm for 7 min, and serum was collected for analysis using human EPO ELISA kits (Stem Cell Technologies; No. 01630) to determine Epo levels following the manufacturer's instructions.

### Alanine aminotransferase/aspartate aminotransferase analysis

Blood was spun down at 5543 *g* for 5 min, serum was collected and analyzed using Beckman AU680 Chemistry Analyzer.

### Immunohistochemistry cleaved caspase-3 and hematoxylin and eosin analysis

Liver and tumor samples were collected from mice at 6 h' post MC3-encapsulated mRNA administration, and fixed in 10% neutral buffered formalin before being dehydrated and paraffin embedded. Tissue blocks were then cut into sections of 5-μm thickness and mounted onto slides. For histopathologic evaluation, one section per tissue was stained by a standard method with hematoxylin and eosin (H&E). Immunohistochemistry (IHC) was performed with cleaved caspase-3 (CC3) antibody (Cell Signaling Technology, Danvers, MA) using the Bond Polymer Refine Detection system followed by hematoxylin and bluing reagent counterstain (Leica Microsystems, Buffalo Grove, IL). The detection system is a biotin-free, polymeric horseradish peroxidase–linker antibody conjugate system run on the Leica Bond RX autostainer.

Images were imaged at 20 × magnification with the Panoramic 250 Flash III whole slide scanner (3DHISTECH, Budapest, Hungary). Image analysis was completed with HALO image analysis software (HALO, Corrales, NM). First, a tissue classifier using a machine learning algorithm was used to identify liver tissue, and next the percentage positive area above a threshold of 3,3′-diaminobenzidine (DAB) intensity was calculated. Final readout was percentage-positive CC3 area over total pixel area.

### Statistical analysis

All results in the article were confirmed with statistical analysis. For each figure, the method deemed appropriate is described in the legend. Statistical significance was defined as *P* value ≤0.05 and determined by Prism using the unpaired, two-tailed *t*-test, or one-way analysis of variance with multiplicity adjustment per the Bonferroni method.

## Results

### Nonspecific biodistribution of protein expression after mRNA delivery

**FIG. 1.**
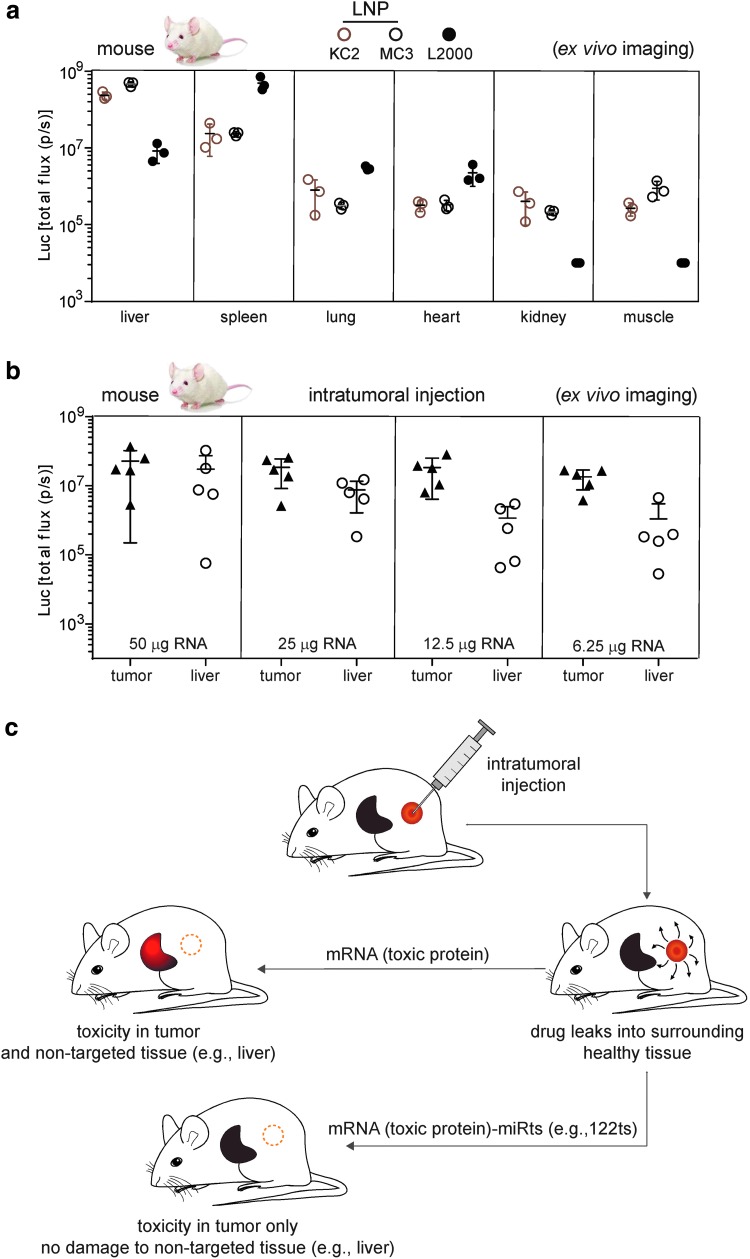
Nonspecific biodistribution of protein expression after mRNA delivery. **(a)** Intravenous delivery of mRNA results in protein expression in multiple tissues. Total flux (photons/s) from indicated organs 6 h after 1-methyl-pseudouridine (m^1^Ψ) Luc mRNA encapsulated in KC2 or MC3 LNPs or in complex with L2000 is intravenously administered to mice. **(b)** Intratumoral delivery of mRNA can lead to protein expression in liver. Total flux (photons/s) from tumor and liver 6 h after m^1^Ψ Luc mRNA encapsulated in MC3 LNP is administered to mice with subcutaneous MC38 tumor. **(c)** Schematic representation to illustrate the possible benefits of using a miRts in mRNAs to dampen off-target expression in mice. Intratumoral injection of an mRNA encoding a toxic protein can trigger cytotoxicity to kill tumor cells but may also damage a nontargeted tissue such as liver. miRts incorporation in the mRNA may restrict expression of toxic proteins to tumor only. Luc, luciferase; LNP, lipid nanoparticle; miRt, microRNA target site.

Current therapeutic mRNA delivery relies predominately on LNPs [1,3]. This delivery method, however, is not tissue specific [8]. For example, while liver accounted for 90%–95% of total luminescence from IV administration to mice of Luc mRNA packaged in either KC2 or MC3 LNPs, 5%–9% originated from the spleen, with lung, muscle, heart, and kidney together contributing 0.3%–0.7% (Fig. 1a). In contrast, mRNA complexed with Lipofectamine 2000 (L2000) yielded highest luminescence in the spleen (Fig. 1a). Thus, neither KC2 or MC3 LNPs nor L2000 fully restrict protein expression to a single tissue.

One way to increase tissue-specific expression is local administration (eg, intratumoral injection). However, factors such as dose, delivery vehicle, and vascular leakage from the tumor can lead to expression outside the tumor microenvironment. Illustrating this is a study where direct injection of MC3-encapsulated Luc mRNA into subcutaneous MC38 tumors in mice resulted in readily detectable Luc activity in the liver (Fig. 1b) across a wide range of mRNA doses.

### Endogenous miRNAs can be exploited to suppress expression from modified mRNAs

One means to reduce expression in off-target tissues is to recruit endogenous miRNAs (Fig. 1c). miRNAs are ∼22 nucleotide noncoding RNAs that negatively regulate protein expression by targeting complementary sequences in the 3′ UTR of mRNAs [9]. Once bound, they can both repress translation and destabilize the mRNA [10,11]. If a miRNA hybridizes with a perfectly complementary target sequence, it can act like a siRNA and initiate mRNA cleavage, leading to even more rapid target clearance [12,13].

Because miRNAs exhibit cell type- and disease-specific expression profiles [14–16], binding sites for miRNAs have been used to suppress protein expression in off-target tissues from viral gene vectors [17–20]. Viral vector-mediated gene expression, however, closely mimics the natural setting in which an mRNA is transcribed from natural nucleotides in the nucleus and exported to the cytoplasm for translation, regulation, and decay. Therapeutic mRNAs, in contrast, are delivered directly to the cytoplasm and often contain modified nucleotides [3].

**FIG. 2.**
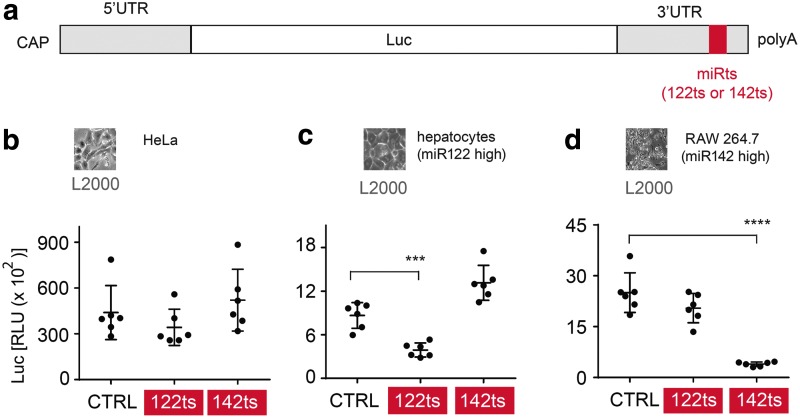
Endogenous miRNAs can be utilized to suppress protein expression from synthetic modified mRNAs in specific cells. **(a)** Schematic representation of Luc mRNA with a miRts in the 3′ UTR. **(b**–**d)** miRts incorporation in modified mRNA leads to suppression of encoded protein in specific cells. RLUs 6 h after L2000-mediated transfection of 1-methyl-pseudouridine (m^1^Ψ) mRNA encoding Luc in **(b)** HeLa, **(c)** primary human hepatocytes, and **(d)** RAW 264.7 macrophages. Each datum in the bar graph is an average of three biological samples, and the error bars represent standard deviation. Luminescence from cells with miRts-containing mRNAs was compared with cells with CTRL mRNA, and *P* values were generated by Prism using the unpaired, two-tailed *t*-test. ***P < 0.001, ****P < 0.0001. miRNA, microRNA; RLU, relative luminescence units; UTR, untranslated region.

To investigate the viability of recruiting endogenous miRNAs to increase the tissue specificity of synthetic therapeutic mRNAs, we incorporated perfectly complementary microRNA target sites (miRts) for either miR122 or miR142 into the 3′ UTR of *in vitro* transcribed mRNA (Fig. 2a). To minimize immune responses, all uridines in the transcribed mRNAs were completely replaced by 1-methyl-pseudouridine (m^1^Ψ) [21]. miR122 is specific to healthy hepatocytes, but is typically repressed in hepatocellular carcinoma (HCC) [22]. miR142 is specific to cells of the hematopoietic lineage [14,16,23]. Thus, a 3′ UTR miR122 target site (122ts) should limit protein expression in healthy hepatocytes but allow it in HCC cells, whereas a 3′ UTR miR142 target site (142ts) should limit protein expression in many antigen presenting cells.

To verify that miRts insertion did not generally compromise mRNA translatability, we confirmed that 122ts or 142ts insertion had no effect on Luc expression in HeLa cells, where miR122 and miR142 are low [24,25] (Fig. 2a, b). In contrast, we observed both cell type- and miRts-specific luminescence decreases in primary human hepatocytes (high miR122, low miR142) and the RAW 264.7 mouse macrophage cell line (low miR122, high miR142) (Fig. 2c, d). Transfection with analogous human Epo mRNAs produced comparable results (data not shown), indicating the generality of the miRts effects.

Finally, to confirm that miRNA-mediated regulation of endogenous mRNAs was unhurt and endogenous miR levels were maintained, we analyzed levels of miR122 itself and several miR122-targeted mRNAs after transfection of miRts mRNAs in hepatocytes. Importantly, no changes were detected in the levels of miR122 or any of the tested miR122 mRNA targets predicted by TargetScan Algorithm [26] (Supplementary Fig. S1). Thus, endogenous miRNAs can be used to limit expression of exogenously delivered mRNAs in specific cells, with minimal perturbation of endogenous mRNA regulation. Because m^1^Ψ had been completely substituted for uridine in all mRNAs used, our data show that chemically modified uridines in and around the miRts are compatible with miRNA-mediated translational suppression.

**FIG. 3.**
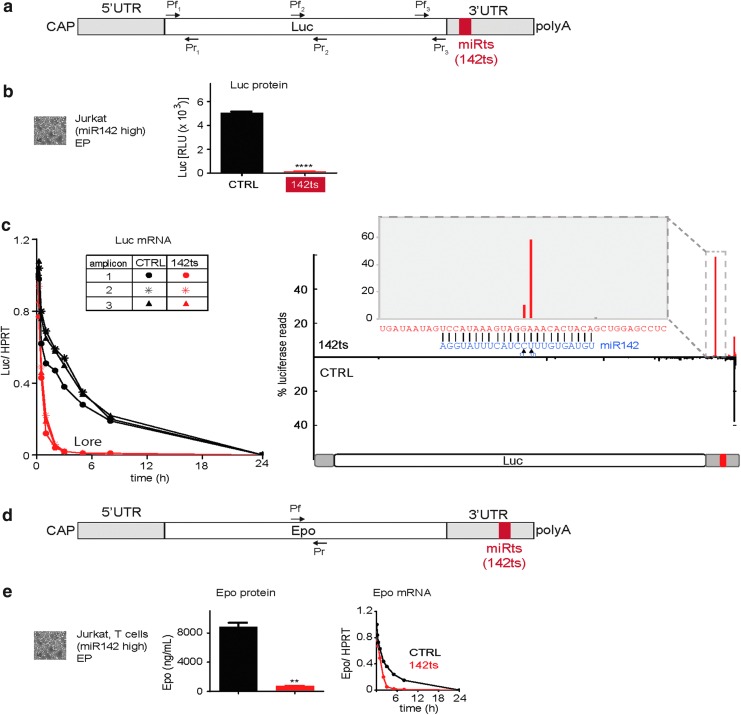
Endogenous miRNAs can act as siRNAs on synthetic modified mRNAs with a miR target site. **(a)** Schematic representation of Luc mRNA with a miRts in the 3′ UTR. **(b**, **c)** Endogenous miRNAs can act as siRNAs on modified mRNA and mediate mRNA cleavage. **(b)** RLUs 24 h after Jurkat cells were EP with 1-methyl-pseudouridine (m^1^Ψ) mRNA encoding Luc. Luminescence from cells with 142ts-containing mRNAs was compared with cells with CTRL mRNA, and *P* values were generated by Prism using the unpaired, two-tailed *t*-test. ****P < 0.0001. **(c)** Luc transcript levels normalized to HPRT transcript at various time points after EP. Luc transcript was assessed using three different primer pairs (Pf1-Pr1, Pf2-Pr2, and Pf3-Pr3) depicted in the schematic representation in **(a)**. All values were normalized to Luc relative to HPRT transcript 5 min after electroporation. Bar graph showing percent total reads obtained across the Luc transcript after 5′-phosphate sequencing from total RNA extracted from cells 30 min post-EP. A zoomed-in view of the *boxed* region in the transcript is shown in the *inset*. miR142 sequence is shown in *blue*. **(d)** Schematic representation of Epo encoding mRNA with a miRts in the 3′ UTR. **(e)** Endogenous miRNAs trigger mRNA decay and protein suppression for modified mRNA for multiple open reading frames. Serum Epo levels 24 h after Jurkat cells were EP with m^1^Ψ mRNA encoding Epo. Epo expression from cells with 142ts-containing mRNAs was compared with cells with CTRL mRNA, and *P* values were generated by Prism using the unpaired, two-tailed *t*-test. **P < 0.01. Epo transcript levels normalized to HPRT at various time points after EP. Epo transcript was assessed using the primer pair (Pf-Pr) depicted in the schematic representation in **(d)**. All values were normalized to Epo relative to HPRT transcript 5 min after electroporation. HPRT, Hypoxanthine phosphoribosyltransferase; siRNA, small interfering RNA; EP, electroporated; Epo, erythropoietin.

We next investigated whether the miRts-mediated suppression was due to mRNA decay. We electroporated control (CTRL; no recognizable miRts) or 142ts Luc mRNA into Jurkat cells, a T cell lymphoma line with high miR142 levels. As observed in macrophages (Fig. 2d), luminescence from the 142ts mRNA electroporated into Jurkat cells was only 3% of that obtained with CTRL mRNA (Fig. 3a, b). Consistent with this, 142ts mRNA was undetectable after 2–3 h (Fig. 3c), whereas ∼20% of CTRL mRNA remained 8 h post-transfection. Parallel results were obtained with 142ts Epo mRNA in Jurkat cells (Fig. 3d, e). We also detected a specific 5′-phosphate-terminated cleavage fragment generated at the miRts (between positions 10 and 11) for 142ts mRNA (Fig. 3c), a hallmark of siRNA-type cleavage reactions. This indicates that endogenous miRNAs can mediate siRNA-like cleavage of chemically modified therapeutic mRNAs. Similar experiments with a different miRts, 126ts, also showed rapid mRNA decay in a miR126-rich endothelial cell line (data not shown).

### miRNA target sites can be used to restrict expression from mRNAs in specific tissues

**FIG. 4.**
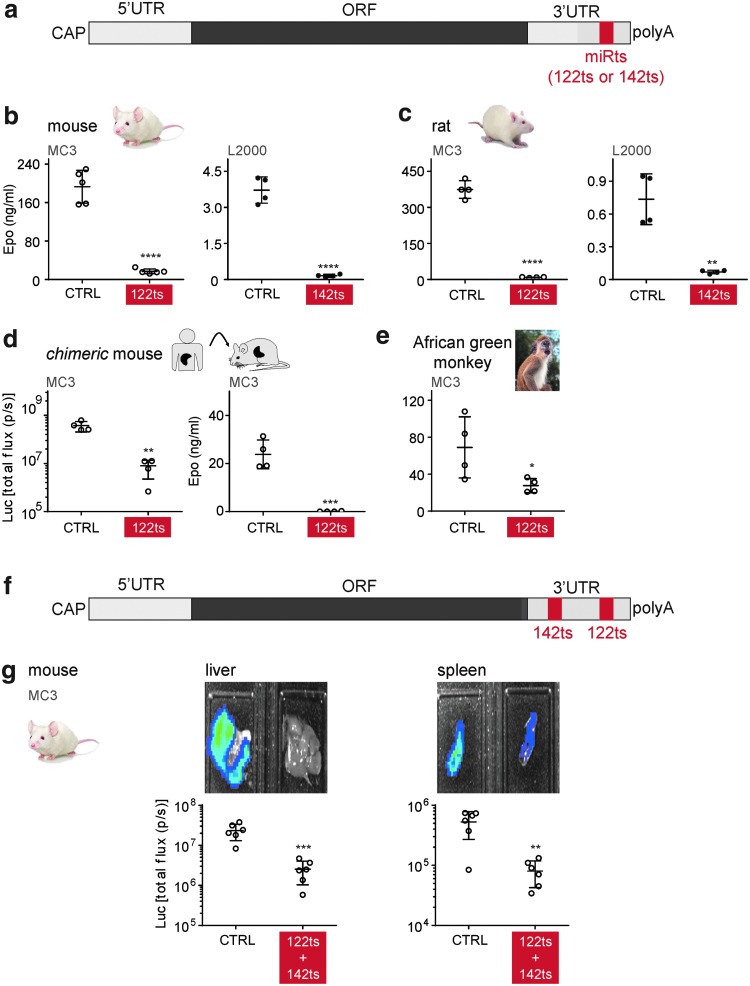
miRNAs can suppress off-target expression in mammals. **(a)** Schematic representation of mRNA with a miRts in the 3′ UTR. **(b**–**d)** miRts incorporation in mRNA enables protein suppression in rodents upon mRNA delivery. Serum Epo levels 6 h after MC3-encapsulated or L2000-complexed 1-methyl-pseudouridine (m^1^Ψ) mRNA encoding Epo was administered intravenously to **(b)** mice or **(c)** rats. **(d)** Total flux (photons/s) and serum Epo levels 6 h after MC3-encapsulated m^1^Ψ mRNAs encoding Luc and Epo were administered intravenously to “chimeric” mice (mouse hepatocytes replaced with human hepatocytes). **(e)** miRts incorporation in mRNA enables protein suppression in nonhuman primates upon mRNA delivery. Serum Epo levels 6 h after MC3-encapsulated m^1^Ψ mRNA encoding Epo was administered intravenously to African green monkey. **(f)** Schematic representation of an mRNA with two different miRts's in the 3′ UTR. **(g)** Incorporation of different miRts's in mRNA enables protein suppression in multiple tissues upon mRNA delivery. Total flux (photons/s) from liver and spleen 6 h after MC3-encapsulated m^1^Ψ mRNA encoding Luc was intravenously administered to mice. Error bars represent standard deviation in the figure. Luminescence/Epo expression generated upon intravenous administration of miRts-containing mRNA was compared with levels with CTRL mRNA, and *P* values were generated by Prism using the unpaired, two-tailed *t*-test. *P < 0.05, **P < 0.01, ***P < 0.001, ****P < 0.0001.

Encouraged by results in tissue culture, we next tested this approach in animals. Upon IV administration of MC3 LNPs in mice, 122ts Epo mRNA yielded 33-fold less serum Epo protein than CTRL Epo mRNA (Fig. 4a, b). This is consistent with MC3 LNPs predominantly driving protein expression in the liver (Fig. 1a). Likewise, incorporation of 142ts led to a 25-fold reduction in Epo expression following IV injection of L2000-complexed mRNAs in mice (Fig. 4b), with L2000 predominantly driving expression in the spleen (Fig. 1a). Comparable results with both 122ts and 142ts mRNAs were obtained in rats (Fig. 4c).

To test if miRts-mediated suppression is likely to work in humans, CTRL and 122ts mRNAs were tested in PhoenixBio (PXB) mice and nonhuman primates. PXB mouse livers are predominantly composed of human hepatocytes. In these “humanized” mice, miRts-mediated knockdown of protein expression was observed with both Luc and Epo reporters (Fig. 4d). African green monkeys also demonstrated protein suppression from miRts-containing mRNAs (Fig. 4e).

We then investigated whether multiple miRts's can be combined to simultaneously downregulate protein expression in multiple tissues. Mice injected with Luc mRNA harboring both a 142ts and a 122ts in the 3′ UTR (Fig. 4f) exhibited 89% and 85% lower luminescence from the liver and spleen, respectively, compared with CTRL Luc mRNA (Fig. 4g). Thus, target sites for different miRNAs can be combined to broaden the tissue repression profile.

### miR122 target sites incorporation in mRNAs encoding toxic proteins (Trojan horse) allow selective elimination of tumor cells

**FIG. 5.**
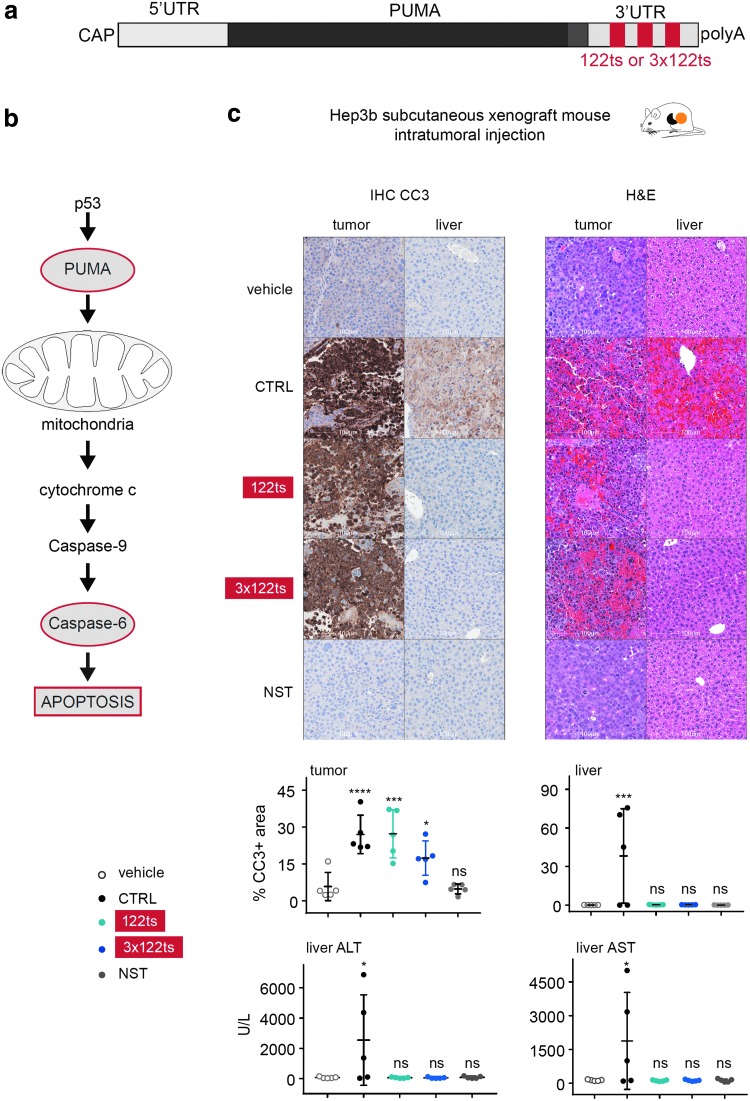
Intratumoral delivery of MC3-encapsulated PUMA-122ts triggers apoptosis in tumor cells while alleviating liver toxicity. **(a)** Schematic representation of PUMA mRNA with miR122 target site(s) (122ts or 3 × 122ts) in the 3′ UTR. **(b)** Schematic representation of PUMA-mediated apoptosis pathway. **(c)** PUMA-122ts triggers apoptosis in tumor cells while alleviating liver toxicity in a Hep3b subcutaneous xenograft mouse model. Representative images from tumor and liver IHC for CC3, and H&E staining 6 h after intratumoral injection of 6.25-μg PUMA mRNA. Quantification of percentage CC3-positive area in tumor and liver, and ALT and AST levels in serum are shown. NST represents an RNA with a similar sequence where all identifiable start codons AUG, CUG, and GUG have been removed. Error bars represent standard deviation in the figure. Levels generated upon administration of LNP-encapsulated mRNA were compared with levels with buffer, and *P* values were generated by Prism using one-way analysis of variance. ns *P* > 0.05, *P < 0.05, ***P < 0.001, ****P < 0.0001. IHC, immunohistochemistry; CC3, cleaved caspase 3; H&E, hematoxylin and eosin; ALT, alanine aminotransferase; AST, aspartate aminotransferase; NST, nonstart RNA.

Having shown the efficacy of miRts's in reducing protein expression in nontarget tissues, we next wanted to explore the possibility of using 122ts to reduce liver toxicity upon intratumoral injection of an mRNA capable of inducing apoptosis (Fig. 5a, b). PUMA is a key mediator of apoptosis known to be upregulated by p53 upon genotoxic stress such as DNA damage [27,28]. Evading apoptosis is an important hallmark of cancer [29], and triggering apoptosis by expressing PUMA can suppress tumorigenesis in certain cancers [30]. Notably, PUMA adenovirus administration has been used to increase the sensitivity of various cancers to anticancer drugs and chemotherapy [27].

To see if we could trigger PUMA-mediated apoptosis selectively in cancer cells using an exogenous mRNA, MC3-encapsulated PUMA mRNAs harboring none, one, or three 122ts's were directly injected into HCC tumors in mice. The PUMA sequence was modified to encode the BH3 domain (Bcl2-homology domain that promotes apoptosis) fused to a peptide aptamer scaffold, Stefin A quadruple mutant, to promote stability of the BH3 structure and enable physiological function [31]. HCC was chosen as it is among the most prevalent cancers with high mortality rates, and therapeutic options increasing survival are currently limited [32]. Importantly, HCC cells are known to have significantly lower miR122 levels than healthy hepatocytes [22]. As a nonfunctional RNA control in the study, the PUMA mRNA sequence was modified to remove all potential start codons (AUG, GUG, and CUG) from the 5′-UTR and coding region to generate a nonstart RNA [33].

All mice receiving CTRL PUMA mRNA exhibited hypoactivity and labored breathing (data not shown). Knowing that intratumorally injected MC3-encapsulated mRNAs can lead to expression in the liver (Fig. 1b), we monitored liver damage by (i) levels of alanine aminotransferase (ALT) and aspartate aminotransferase (AST), enzymes that are spilled into the blood upon liver injury; (ii) H&E staining; and (iii) IHC of CC3, the major downstream effector of Caspase-mediated apoptosis [34]. Liver toxicity was manifest in serum, and liver tissue sections taken 6 h after the initial injection of CTRL PUMA mRNA into the tumor (Fig. 5c). In sharp contrast, no appreciable behavioral differences or liver toxicities were observed in mice receiving the 122ts or 3 × 122ts PUMA mRNA. Tumor cells, however, exhibited similar degrees of damage in both the CTRL and 122ts groups (Fig. 5c). These findings were recapitulated at a 4 × higher mRNA dose (Supplementary Fig. S2a, b). Taken together, our results demonstrate the feasibility of using mRNAs with miRts's to encode toxic proteins that induce apoptosis of cancer cells without damaging healthy hepatocytes.

### miR122 target sites completely alleviate liver damage after systemic delivery of a Trojan horse mRNA

**FIG. 6.**
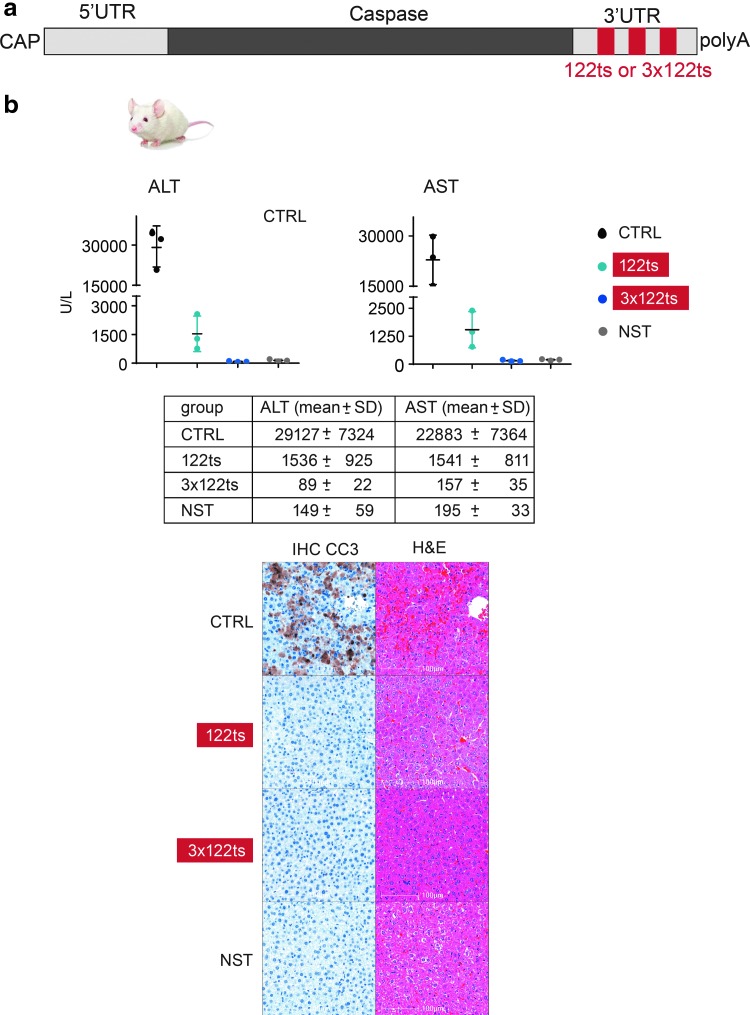
miR122 target site incorporation alleviates liver damage from systemic delivery of MC3-encapsulated Caspase mRNA. **(a)** Schematic representation of Caspase mRNA with miR122 target site(s) (122ts or 3 × 122ts) in the 3′ UTR. **(b)** Intravenous delivery of MC3-encapsulated Caspase-122ts alleviates liver damage from Caspase mRNA. Serum levels of ALT and AST 6 h after intravenous administration of MC3-encapsulated Caspase mRNA in mice. Error bars represent standard deviation in the figure. Representative images from liver IHC for CC3, and H&E staining. NST represents an RNA with a similar sequence where all identifiable start codons AUG, CUG, and GUG have been removed (NST). The *P* values are not reported in this experiment because of the lower *n*. The mean and standard deviation for each group are shown in the table.

Finally, we asked whether a 122ts could suppress liver toxicity from an mRNA encoding a lethal protein even under conditions where the bulk of protein expression is driven from the liver (eg, IV injection of MC3-encapsulated mRNA, Fig. 1a). To do so, mice were intravenously administered MC3-encapsulated reversed-Caspase-6 mRNA (Fig. 6a). Reversed-Caspase-6 is a constitutively active form of Caspase and causes apoptosis [35]. Mice receiving CTRL mRNA exhibited severe toxicity as measured by a sharp rise in serum ALT/AST levels, IHC CC3, and H&E staining, whereas mice dosed with 122ts or 3 × 122ts reversed-Caspase-6 mRNA exhibited reduced or no liver toxicity, respectively (Fig. 6b). Thus, we successfully ameliorated liver damage from a toxic payload, even with a delivery route known to predominantly drive protein expression in liver.

## Discussion

Here, we have demonstrated that miRts' incorporation into synthetic, modified mRNAs provides a means to mitigate off-target expression, an oft-raised concern in the field of mRNA therapeutics [8,36]. Insertion of a single or multiple perfectly complementary miRts's into the 3′ UTR substantially suppressed protein expression in cells harboring elevated levels of the corresponding miRNA in cell culture as well as *in vivo* in both rodents and primates. miR-mediated suppression is accompanied by an siRNA-type cleavage event at the complementary target sequence in the mRNA. To our knowledge, this is the first demonstration that RISC-mediated cleavage is refractory to nucleobase modifications in the targeted nucleic acid.

To date, all examples of mRNA therapeutics have encoded either a protein to trigger an immune response or an endogenous protein to address a deficiency [1,3]. The ability to suppress off-target expression using miRts's now opens the possibility to express one or more toxic cargoes specifically in disease cells without harming healthy cells that may receive the same mRNA payload. Further, the transient nature of therapeutic mRNAs makes them potentially more attractive as a means to deliver toxic payloads than viral systems, expression from which can persist for months to years.

The miR strategy presented here enhances the repertoire of diseases potentially targetable with mRNA therapeutics by enabling selective elimination of diseased cells, without incurring systemic toxicity. In addition to the benefits of using 122ts to reduce damage from toxic cargoes in miR122-high hepatocytes, miRts's complementary to miRNAs dominant in antigen presenting cells (eg, miR142) could become especially important in mitigating potential immune responses triggered upon intracellular expression of a novel protein from therapeutic mRNAs. In combination with targeted LNP delivery and modified nucleotides, we anticipate that miRts's will be valuable in enabling selective expression of therapeutic proteins in efficacious doses from synthetic mRNAs in specific tissues.

## Supplementary Material

Supplemental data

Supplemental data

Supplemental data

Supplemental data
