# Supplementary material for: MicroRNAs Enable mRNA Therapeutics to Selectively Program Cancer Cells to Self-Destruct
Source: Nucleic Acid Ther. 2018 Sep 24;28(5):285–96. doi: 10.1089/nat.2018.0734 (PMC6157376; doi:10.1089/nat.2018.0734)
Supplement: Supplemental data [file Supp_Table1.pdf]

Supplementary Table S1. List of Sequences Used

5′ UTR
GGGAAATAAGAGAGAAAAGAAGAGTAAGAAGAAATATAAGAGCCACC

3′ UTR (control)
TGATAATAGGCTGGAGCCTCGGTGGCCATGCTTCTTGCCCCTTGGGCCTCCCCCCAGCCCCTCCTCCCCTTCCT
GCACCCGTACCCCCGTGGTCTTTGAATAAAGTCTGAGTGGGCGGC

3′ UTR (122ts).
TGATAATAGGCTGGAGCCTCGGTGGCCATGCTTCTTGCCCCTTGGGCCTCCCCCCAGCCCCTCCTCCCCTTCCT
GCACCCGTACCCCC**CAAACACCATTGTCACACTCCA**GTGGTCTTTGAATAAAGTCTGAGTGGGCGGC

3′ UTR (142ts)
TGATAATAGGCTGGAGCCTCGGTGGCCATGCTTCTTGCCCCTTGGGCCTCCCCCCAGCCCCTCCTCCCCTTCCT
GCACCCGTACCCCC**TCCATAAAGTAGGAAACACTACA**GTGGTCTTTGAATAAAGTCTGAGTGGGCGGC

3′ UTR (122ts +142ts)
TGATAATAG**TCCATAAAGTAGGAAACACTACA**GCTGGAGCCTCGGTGGCCATGCTTCTTGCCCCTTGGGCC
CAAACACCATTGTCACACTCCATCCCCCCAGCCCCTCCTCCCCTTCCTGCACCCGTACCCCCGTGGTCTTT
GAATAAAGTCTGAGTGGGCGGC

3′ UTR (142ts) (RLM-RACE)
TGATAATAG**TCCATAAAGTAGGAAACACTACA**GCTGGAGCCTCGGTGGCCATGCTTCTTGCCCCTTGGGCCT
CCCCCCAGCCCCTCCTCCCCTTCCTGCACCCGTACCCCCGTGGTCTTTGAATAAAGTCTGAGTGGGCGGC

3′ UTR (3X_122ts)
TGATAATAG**CAAACACCATTGTCACACTCCA**GCTGGAGCCTCGGTGGCCATGCTTCTTGCCCCTTGGGCC
CAAACACCATTGTCACACTCCATCCCCCCAGCCCCTCCTCCCCTTCCTGCACCCGTACCCCC**CAAACAC**
CATTGTCACACTCCAGTGGTCTTTGAATAAAGTCTGAGTGGGCGGC

Luc ORF
ATGGAAGATGCGAAGAACATCAAGAAGGGACCTGCCCCGTTTTACCCTTTGGAGGACGGTACAGCAGGAGA
ACAGCTCCACAAGGCGATGAAACGCTACGCCCTGGTCCCCGGAACGATTGCGTTTACCGATGCACATATT
GAGGTAGACATCACATACGCAGAATACTTCGAAATGTCGGTGAGGCTGGCGGAAGCGATGAAGAGATATG
GTCTTAACACTAATCACCGCATCGTGGTGTGTTCGGAGAACTCATTGCAGTTTTTCATGCCGGTCCTTGGAG
CACTTTTCATCGGGGTCGCAGTCGCGCCAGCGAACGACATCTACAATGAGCGGGAACTCTTGAATAGCATG
GGAATCTCCCAGCCGACGGTCGTGTTTGTCTCCAAAAAGGGGCTGCAGAAAATCCTCAACGTGCAGAAG
AAGCTCCCCATTATTCAAAAGATCATCATTATGGATAGCAAGACAGATTACCAAGGGTTCCAGTCGATGT
ATACCTTTGTGACATCGCATTTGCCGCCAGGGTTTAACGAGTATGACTTCGTCCCCGAGTCATTTGACAGA
GATAAAACCATCGCGCTGATTATGAATTCCTCGGGTAGCACCGGTTTGCCAAAGGGGGTGGCGTTGCCCC
ACCGCACTGCTTGTGTGCGGTTCTCGCACGCTAGGGATCCTATCTTTGGTAATCAGATCATTCCCGACACAG
CAATCCTGTCCGTGGTACCTTTTCATCACGGTTTTGGCATGTTCACGACTCTCGGCTATTTGATTTGCGGTT
TCAGGGTCGTACTTATGTATCGGTTCGAGGAAGAACTGTTTTTGAGATCCTTGCAAGATTACAAGATCCAG
TCGGCCCTCCTTGTGCCAACGCTTTTCTCATTCTTTGCGAAATCGACACTTATTGATAAGTATGACCTTTCC
AATCTGCATGAGATTGCCTCAGGGGGAGCGCCGCTTAGCAAGGAAGTCGGGGAGGCAGTGGCCAAGCGC
TTCCACCTTCCCGGAATTCGGCAGGGATACGGGCTCACGGAGACAACATCCGCGATCCTTATCACGCCCGA
GGGTGACGATAAGCCGGGAGCCGTCGGAAAAGTGGTCCCCTTCTTTGAAGCCAAGGTCGTAGACCTCGA
CACGGGAAAAACCCTCGGAGTGAACCAGAGGGGCGAGCTCTGCGTGAGAGGGCCGATGATCATGTCAGG
TTACGTGAATAACCCTGAAGCGACGAATGCGCTGATCGACAAGGATGGGTGGTTGCATTCGGGAGACAT
TGCCTATTGGGATGAGGATGAGCACTTCTTTATCGTAGATCGACTTAAGAGCTTGATCAAATACAAAGGC
TATCAGGTAGCGCCTGCCGAGCTCGAGTCAATCCTGCTCCAGCACCCCAACATTTTCGACGCCGGAGTG
GCCGGGTTGCCCGATGACGACGCGGGTGAGCTGCCCAGCGCGGCCGTGGTAGTCCTCGAACATGGGAAAACA
ATGACCGAAAAGGAGATCGTGGACTACGTAGCATCACAAGTGACGACTGCGAAGAAACTGAGGGGAGGG
GTAGTCTTTGTGGACGAGGTCCCGAAAGGCTTGACTGGGAAGCTTGACGCTCGCAAAATCCGGGAAATCCT
GATTAAGGCAAAGAAAGGCGGGAAAATCGCTGTC

Epo ORF
ATGGGAGTGCACGAGTGTCCCGCGTGGTTGTGGTTGCTGCTGTCGCTCTTGAGCCTCCCACTGGGACTGCCTG
TGCTGGGGGCACCACCCAGATTGATCTGCGACTCACGGGTACTTGAGAGGTACCTTCTTGAAGCCAAAGA
AGCCGAAAACATCACAACCGGATGCGCCGAGCACTGCTCCCTCAATGAGAACATTACTGTACCGGATACA
AAGGTCAATTTCTATGCATGGAAGAGAATGGAAGTAGGACAGCAGGCCGTCGAAGTGTGGCAGGGGCTCG
CGCTTTTGTCGGAGGCGGTGTTGCGGGGTCAGGCCCTCCTCGTCAACTCATCACAGCCGTGGGAGCCCCTC
CAACTTCATGTCGATAAAGCGGTGTCGGGGCTCCGCAGCTTGACGACGTTGCTTCGGGCTCTGGGCGCAC
AAAAGGAGGCTATTTCGCCGCCTGACGCGGCCTCCGCGGCACCCCTCCGAACGATCACCGCGGACACGTT
TAGGAAGCTTTTTAGAGTGTACAGCAATTTCCTCCGCGGAAAGCTGAAATTGTATACTGGTGAAGCGTGTA
GGACAGGGGATCGC

PUMA ORF
ATGATCCCCCGCGAGGAGCAGTGGGCGCGCGAGATCGGTGCACAACTGCGCAGAATGGCGGATGATCTGAA
TGCTCAGTATGAGCGTAGGGGACTGTCCGAGGCTAAACCAGCCACTCCTGAGATTCAAGAAATTGTGGAT
AAAGTAAAACCTCAGCTGGAGGAGAAAACTAATGAAACATATGGGAAACTCGAAGCCGTGCAGTACAA
GACGCAAGTTCTGGCGAGCACCAATTACTATATCAAGGTGCGCGCAGGCGATAATAAATATATGCACCT
GAAAGTATTTAACGGGCCGCCAGGGCAGAATGCAGATAGAGTGCTGACAGGATATCAGGTCGATAAAAA
TAAGGACGATGAACTGACGGGTTTCGATTACAAAGATGACGATGACAAA

(*continued*)

Caspase ORF

ATGGTAGAAATAGATGCAGCCTCCGTTTACACGCTGCCTGCTGGAGCTGACTTCCTCATGTGTTACTCTGTT
GCAGAAGGATATTATTCTCACCGGGAAACTGTGAACGGCTCATGGTACATTCAAGATTTGTGTGAGATG
TTGGGAAAATATGGCTCCTCCTTAGAGTTCACAGAACTCCTCACACTGGTGAACAGGAAAGTTTCTCAGC
GCCGAGTGGACTTTTGCAAAGACCCAAGTGCAATTGGAAAGAAGCAGGTTCCCTGTTTTGCCTCAATGCT
AACTAAAAAGCTGCATTTCTTTCCAAAATCTAATCTCGAGCACCACCACCACCACCACGTTGAAATTGAT
GGGGGATCCCCCATGAGCTCGGCCTCGGGGCTCCGCAGGGGGCACCCGGCAGGTGGGGAAGAAAACAT
GACAGAAACAGATGCCTTCTATAAAAGAGAAATGTTTGATCCGGCAGAAAAGTACAAAATGGACCACAG
GAGGAGAGGAATTGCTTTAATCTTCAATCATGAGAGGTTCTTTTGGCACTTAACACTGCCAGAAAGGCGG
GGCACCTGCGCAGATAGAGACAATCTTACCCGCAGGTTTTCAGATCTAGGATTTGAAGTGAAATGCTTTA
ATGATCTTAAAGCAGAAGAACTACTGCTCAAAATTCATGAGGTGTCAACTGTTAGCCACGCAGATGCCGA
TTGCTTTGTGTGTGTCTTCCTGAGCCATGGCGAAGGCAATCACATTTATGCATATGATGCTAAAATCGAAA
TTCAGACATTAACTGGCTTGTTCAAAGGAGACAAGTGTCACAGCCTGGTTGGAAAACCCAAGATATTTAT
CATCCAGGCATGTCGGGGAAACCAGCACGATGTGCCAGTCATTCCTTTGGATGTAGTAGAT

NST

AGGGTAGAAATAGATCCAGCCTCCGTTTACACGTTGCTTGTTGGAGTTGACTTCCTCTTGTCTTACTTTGTTG
CAGAAGGATATTATTCTCACCGGGAAATTGTCAACGGCTCATTGTACATTCAAGATTTGTCTCAGATCTTG
GGAAAATAGCGCTCCTCCTTAGAGTTCACAGAACTCCTCACATTGGTCAACAGGAAAGTTTCTCAGCGCC
GAGTCGACTTTTGCAAAGACCCAAGTCCAATTGGAAAGAAGCAGGTTCCTTGTTTTGCCTCATTGCTAAC
TAAAAAGTTGCATTTCTTTCCAAAATCTAATCTCGAGCACCACCACCACCACCACGTTGAAATTGATTGG
GGATCCCCCATTAGCTCGGCCTCGGGGCTCCGCAGGGGGCACCCGGCAGGTCGGGAAGAAAACATTACA
GAAACAGATTCCTTCTATAAAAGAGAAATCTTTGATCCGGCAGAAAAGTACAAAATCGACCACAGGAGG
AGAGGAATTGCTTTAATCTTCAATCATCAGAGGTTCTTTTGGCACTTAACATTGCCAGAAAGGCGGGGCA
CTTGCGCAGATAGAGACAATCTTACCCGCAGGTTTTCAGATCTAGGATTTGAAGTCAAATCCTTTAATCAT
CTTAAAGCAGAAGAACTATTGCTCAAAATTCATCAGGTCTCAATTGTTAGCCACGCAGATCCCGATTGCTT
TGTCTCTCTCTTCTTGAGCCATCGCGAAGGCAATCACATTTATCCATATCATCCTAAAATCGAAATTCAGAC
ATTAATTGGCTTGTTCAAAGGAGACAAGTCTCACAGCTTGGTTGGAAAACCCAAGATATTTATCATCCAG
GCATCTCGGGGAAACCAGCACGATTTGCCAGTCATTCCTTTGGATCTAGTAGAT

Micro RNA target sites are indicated in *bold*.

Luc, luciferase; Epo, erythropoietin; NST, non-start RNA; ORF, open reading frame; UTR, untranslated region.
