# Supplementary material for: MicroRNAs Enable mRNA Therapeutics to Selectively Program Cancer Cells to Self-Destruct
Source: Nucleic Acid Ther. 2018 Sep 24;28(5):285–96. doi: 10.1089/nat.2018.0734 (PMC6157376; doi:10.1089/nat.2018.0734)
Supplement: Supplemental data [file Supp_Table2.pdf]

Supplementary Table S2. List of Primers Used

Primer	*Sequence*
Pforward	TGGACCCTCGTACAGAAGCTAATACG
Preverse	$(T)_{100}$GCCGCCCACTCAGACTTTATTCAAAGACCAC
Pf_1(Luc)	GAAGATGCGAAGAACATCAAGAAGGGAC
Pr_1(Luc)	GGACCAGGGCGTAGCGTTTCATCGCC
Pf_2(Luc)	GTGCGGTTCTCGCACGCTAGGGATCCTATC
Pr_2(Luc)	CCGCAAATCAAATAGCCGAGAGTCGTGAAC
Pf_3(Luc)	CCAACATTTTCGACGCCGGAGTGGCCG
Pr_3(Luc)	CCTTTTCGGTCATTGTTTTCCCATGTTCGAG
Pf (Epo)	TGCATGGAAGAGAATGGAAGTAGGACAGC
Pr(Epo)	GATGAGTTGACGAGGAGGGCCTGAC
HPRT_f	CAATGCAGACTTTGCTTTCCTTGGTCAGGCAG
HPRT_r	TCCAACACTTCGTGGGGTCCTTTTCACCAGC
3′-oligo	TTCAGACGTGTGCTCTTCCGATCTCCACTCAGACTTTATTCAAAGACCAC

HPRT, Hypoxanthine phosphoribosyltransferase.
