# Supplementary material for: MicroRNAs Enable mRNA Therapeutics to Selectively Program Cancer Cells to Self-Destruct
Source: Nucleic Acid Ther. 2018 Sep 24;28(5):285–96. doi: 10.1089/nat.2018.0734 (PMC6157376; doi:10.1089/nat.2018.0734)
Supplement: Supplemental data [file Supp_Fig1.pdf]

Supplementary Data

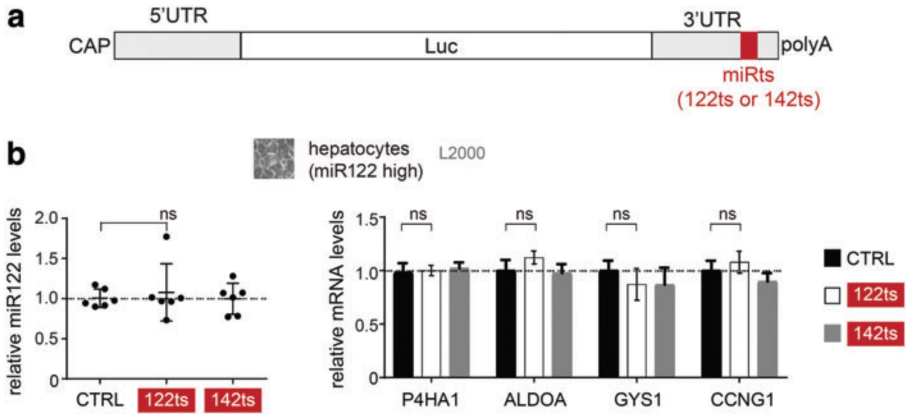

SUPPLEMENTARY FIG. S1. Administration of 122ts-containing-mRNA does not hurt endogenous miR122 levels or miR122-mediated mRNA regulation in hepatocytes. **(a)** Schematic representation of Luc mRNA with a miRts in the 3′ UTR. **(b)** miR122ts incorporation in modified mRNA does not hurt endogenous miR-regulation pathways. *Left panel*. Relative miR122 levels in hepatocytes 6 h after transfection with mRNA encoding Luc. All values were normalized to Z30 RNA. miR122 levels compared with cells that received CTRL mRNA with no miRts are shown. *Right panel*. Relative mRNA levels of known miR122-targeted mRNAs in hepatocytes 6 h after transfection with mRNA encoding Luc. All values were normalized to GAPDH RNA. mRNA levels compared with cells that received CTRL mRNA with no miRts are shown. Levels detected upon administration of 122ts mRNA were compared with levels with CTRL, and *P* values were generated by Prism using one-way analysis of variance. Luc, luciferase; miRt, microRNA target site; UTR, untranslated region.
