# Supplementary material for: MicroRNAs Enable mRNA Therapeutics to Selectively Program Cancer Cells to Self-Destruct
Source: Nucleic Acid Ther. 2018 Sep 24;28(5):285–96. doi: 10.1089/nat.2018.0734 (PMC6157376; doi:10.1089/nat.2018.0734)
Supplement: Supplemental data [file Supp_Fig2.pdf]

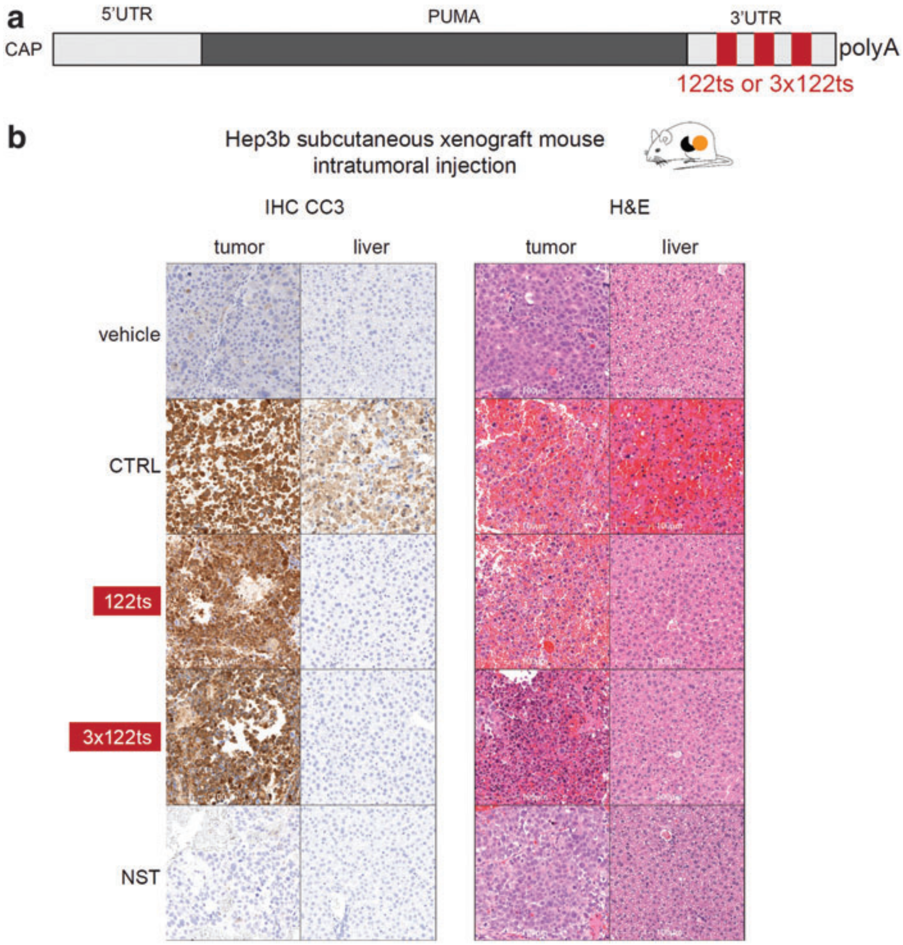

SUPPLEMENTARY FIG. S2. Intratumoral delivery of MC3-encapsulated PUMA-122ts triggers apoptosis in tumor cells while alleviating liver toxicity. **(a)** Schematic representation of PUMA mRNA with miR122 target site(s) (122ts or 3 × 122ts) in the 3′ UTR. **(b)** PUMA-122ts triggers apoptosis in tumor cells while alleviating liver toxicity in a Hep3b subcutaneous xenograft mouse model. Representative images from tumor and liver IHC for CC3, and H&E staining 6 h after intratumoral injection of 25-μg PUMA mRNA. NST represents an RNA with a similar sequence where all identifiable start codons AUG, CUG, and GUG have been removed. IHC, immunohistochemistry; CC3, cleaved caspase 3; H&E, hematoxylin and eosin; NST, nonstart RNA.
